# Body Fatness and Markers of Thyroid Function among U.S. Men and Women

**DOI:** 10.1371/journal.pone.0034979

**Published:** 2012-04-12

**Authors:** Cari M. Kitahara, Elizabeth A. Platz, Paul W. Ladenson, Alison M. Mondul, Andy Menke, Amy Berrington de González

**Affiliations:** 1 Department of Epidemiology, Johns Hopkins Bloomberg School of Public Health, Baltimore, Maryland, United States of America; 2 Division of Cancer Epidemiology and Genetics, National Cancer Institute, National Institutes of Health, Rockville, Maryland, United States of America; 3 Division of Endocrinology and Metabolism, Department of Medicine, Johns Hopkins Medical Institutions, Baltimore, Maryland, United States of America; Johns Hopkins Bloomberg School of Public Health, United States of America

## Abstract

**Background:**

We evaluated the association of central versus overall adiposity on levels of thyroid stimulating hormone (TSH), free triiodothyronine (fT_3_), and free thyroxine (fT_4_) among euthyroid subjects taken from a cross-sectional, representative sample of the adult non-institutionalized U.S. population.

**Methods:**

The National Health and Nutrition Examination Survey 2007–2008 included 1,623 men and 1,491 women who were 20 years and older, with no history of thyroid or liver disease, kidney failure, diabetes, or thyroid function-altering prescription medication use (based on self-report), and having TSH, fT_3_, and fT_4_ levels between 0.5–4.49 mIU/L, 2.5–3.9 pg/mL, and 0.6–1.6 ng/dL, respectively. Associations between body mass index (BMI) and waist circumference (measures of overall and central adiposity, respectively) and TSH, fT_3_, and fT_4_ levels were estimated using multivariable linear regression models stratified by sex and adjusted for age, race, smoking status, and alcohol intake.

**Results:**

An increase in serum TSH levels was observed for every 1-quartile increase in BMI in euthyroid men (3.8% [95% CI 0.8%, 6.8%]) and euthyroid women (4.0% [95% CI 1.6%, 6.5%]). Similar, albeit slightly weaker, associations were observed with waist circumference. We also found increases in fT_3_ levels with every 1-quartile increase in BMI (1.0% in men and 1.3% in women) and waist circumference (1.2% in men and 1.2% in women). No associations were observed with fT_4._

**Conclusions:**

Our results provide support that BMI and waist circumference are positively associated with levels of serum TSH and f T_3_ but not fT_4_ among euthyroid adults. Longitudinal studies are needed to define the temporality of these associations and their potential health implications.

## Introduction

The growing prevalence of overweight and obesity is becoming an increasingly important health problem throughout the world [1] as excess body weight is a known risk factor for mortality [2] and a multitude of diseases, including diabetes [3], [4], hypertension [4], heart disease [4], ischemic stroke [5], and several types of cancer [6]. While weight change is largely attributable to an imbalance in energy intake and expenditure [1], it is also a well-recognized and common manifestation of overt thyroid dysfunction due to regulation of resting energy expenditure (REE) by thyroid hormones [7]–[8].

The potential mechanisms underlying the association between body fat and thyroid function are less clear among euthyroid individuals, in whom it is possible that weight change could precede subsequent changes in thyroid hormone (triiodothyronine [T_3_] and thyroxine [T_4_]) and thyroid stimulating hormone (TSH) levels. Several cross-sectional studies have shown associations of body fat with higher serum TSH [9]–[16] and T_3_ [17] and lower T_4_ [15], [16], [18], [19] among euthyroid individuals, though there have been some inconsistencies [12]–[14], [17]–[20]. However, many of these studies were limited due to small sample sizes (<500 participants) [13]–[15], [17], [20]; lack of adjustment for important covariates associated with TSH, such as cigarette smoking [12]–[15], [20], [21]; or only examined the relationship in special sub-groups such as overweight or obese participants [14], [17] or individuals with a medical history of thyroid disease [20], which limits the generalizability of the results. There is also conflicting evidence on the association between central as opposed to overall adiposity and TSH levels among euthyroid adults [13], [17]. Thus, it remains unclear whether hormonal and metabolic alterations common to central adiposity (e.g., insulin resistance) may influence, or be influenced by, thyroid status.

We investigated the association between body fatness, using body mass index (BMI) and waist circumference as measures of overall and central adiposity, respectively, and serum TSH, fT_3_, and fT_4_ as measures of thyroid function among euthyroid adult men and women using data from a nationally representative cross-sectional study in the U.S., a population that includes a wide range of body fatness as well as thyroid function.

## Methods

### Study population

The National Health and Nutrition Examination Survey (NHANES) program began in 1960 as a series of cross-sectional surveys of the U.S. civilian, non-institutionalized population conducted for the purposes of obtaining nationally-representative estimates of health and nutritional status. The program employs a stratified, multistage probability sampling design with oversampling of certain income, age, and race/ethnicity subgroups. Beginning in 1999, surveys were released in 2-year cycles. The data collection process for this survey has been previously described in detail [22]. Study protocols for NHANES were approved by the institutional review board at the National Center for Health Statistics. All participants provided written informed consent. Reports from NHANES indicate that the general U.S. population is nutritionally iodine sufficient [23].

The eligible study population was restricted to 5,935 individuals ages 20 and older who participated in NHANES 2007–2008. We further excluded participants who were pregnant (n = 57); had a self-reported medical history of thyroid disease (n = 552); reported taking prescription medications that may influence thyroid function (n = 175); reported having a medical condition that may influence thyroid function, including diabetes, kidney, and liver disease (n = 155); reported having general “poor health” (n = 135); had missing data on BMI (n = 211), waist circumference (n = 155), levels of TSH (n = 268) or free T_3_ or free T_4_ (n = 3); or had extreme values for BMI (<15 or >50 kg/m^2^, n = 17). We also excluded participants with TSH, fT_3_, and fT_4_ outside the ranges of 0.5–4.49 mIU/L (n = 282), 2.5–3.9 pg/mL (n = 144), and 0.6–1.6 ng/dL (n = 55), respectively [24]. After these exclusions, a total of 1,623 men and 1,491 women were eligible for this analysis.

### Exposure assessment and classification

A team consisting of physicians, health and dietary interviewers, and medical and health technicians conducted in-home interviews and performed physical examinations from specially-designed and equipped mobile centers. Interview items included age, sex, education, race/ethnicity, pregnancy status, medical history of thyroid disease (including thyroid cancer), use of prescription medications, cigarette smoking, and alcohol intake. Interviewers also obtained information on prescription medications from labels on containers brought to the interview by the participants. Height, weight, and waist circumference were measured in mobile examination centers by trained health technicians using standardized protocols and regularly-calibrated equipment. Waist circumference was measured at minimal respiration with a flexible anthropometric tape positioned parallel to the floor directly above the iliac crest. Study staff previously reviewed and deleted unusual or erroneous values from the 2007–2008 datasets.

### Laboratory measurements

Participants who did not meet any of the exclusion criteria for providing blood samples (i.e., hemophilia, recent recipient of chemotherapy, certain medical symptoms) were eligible for thyroid function assessment between 2007 and 2008, including the measurement of TSH, fT_3_, fT_4_, and thyroid autoantibodies. Serum samples were analyzed for TSH (Access HYPERsensitive human thyroid-stimulating hormone [hTSH] assay; Beckman Coulter, Fullerton, CA), fT_3_ (Access free T_3_ assay; Beckman Coulter, Fullerton, CA), fT_4_ (Access free T_4_ assay; Beckman Coulter, Fullerton, CA), thyroperoxidase autoantibodies (TPOab; Access TPO antibody assay, Beckman Coulter, Fullerton, CA), and thyroglobulin antibodies (TgAb; Access thyroglobulin antibody assay, Beckman Coulter, Fullerton, CA) at the University of Washington Medical Center, Department of Laboratory Medicine, Immunology Division. The distribution of these measures in the study population are shown in **Table 1**. The reference ranges for TSH, fT_3_, fT_4_, TPOab, TgAb were 0.34–5.60 mIU/L, 2.5–3.9 pg/mL, 0.6–1.6 ng/dL, 0–9.0 IU/mL, and 0–4.0 IU/mL, respectively [24]. Geometric means, medians, 25^th^ percentiles, and 75^th^ percentiles for these hormones, as well as thyroid autoantibodies, among all adults (ages ≥20) prior to the study-specific exclusions are shown in **Table S1**. Inter-assay coefficients of variation ranged from 4.4–6.5%, 3.5–5.6%, 2.8–9.3%, 6.3–7.6%, and 6.1–9.3% for TSH, fT_3_, fT_4_, TPOab, and TgAb, respectively. The quality control and quality assurance protocols used in NHANES meet the 1988 Clinical Laboratory Improvement Act standards.

**Table 1:** Geometric means for TSH, fT_3_, and fT_4_ levels by select characteristics of male and female participants, NHANES 2007–2008.

	No. participants	Hormone concentrations		
		TSH (mIU/L)	fT_3_ (pg/mL)	fT_4_ (ng/dL)
**Sex**				
Men	1,623	1.58	3.26	0.77
Women	1,491	1.61	3.08	0.76
**Age (years)**				
20–34	851	1.43	3.27	0.77
35–49	911	1.57	3.18	0.75
50–64	751	1.72	3.13	0.76
65–80	601	1.85	2.99	0.79
**Race/ethnicity**				
Non-Hispanic white	1,487	1.66	3.16	0.76
Hispanic	925	1.49	3.28	0.77
Non-Hispanic black	565	1.30	3.13	0.75
Other	137	1.55	3.19	0.81
**BMI (kg/m^2^): WHO categories** a				
15–18.4 (underweight)	49	1.59	3.13	0.80
18.5–24.9 (normal)	932	1.48	3.13	0.77
25–29.9 (overweight)	1,158	1.63	3.18	0.76
30–34.9 (obese class I)	598	1.69	3.21	0.76
35–50 (obese class II & III)	377	1.63	3.21	0.76
**Waist circumference (cm)**				
<90	1,044	1.51	3.13	0.76
90–99	839	1.61	3.18	0.76
100–109	666	1.64	3.21	0.76
≥110	565	1.69	3.22	0.76
**Smoking status** b				
Never	1,681	1.62	3.15	0.76
Former	733	1.66	3.14	0.76
Current	699	1.46	3.25	0.76
**Alcohol intake (drinks/day)** b				
None	1,085	1.61	3.15	0.77
1–2	1,061	1.65	3.13	0.76
>2	809	1.52	3.25	0.76

a World Health Organization classification of adult underweight, normal-weight, overweight, and obesity according to BMI [37].

b Does not include missing values.

### Statistical methods

The complex, multistage, probability sampling survey design data from NHANES 2007–2008 were analyzed using the survey design commands in Stata version 9.2 (StataCorp, College Station, TX). All descriptive statistics and regression analyses incorporated the mobile examination center exam two-year sampling weights. We examined the sex-specific associations of body fatness measures (BMI and waist circumference; independent variables) with TSH, fT_3_, and fT_4_ (dependent variables) using multivariable linear regression, adjusting for age, race (non-Hispanic white, non-Hispanic black, Hispanic, African American, other), smoking status (never, former, current, missing), and alcohol intake (over the past 12 months: none, 1 drink/day, 2 drinks/day, >2 drinks/day, missing). In order to be directly comparable, the body fatness measures were categorized into quartiles (see **Table S2** for quartile cutpoints), and these quartiles were modeled as continuous variables. TSH, fT_3_, and fT_4_ concentrations were natural-log transformed to more closely approximate normal distributions and then back-transformed so that the beta coefficients from the models represent a % increase in TSH, fT_3_, or fT_4_ for a 1-quartile increase in the anthropometric variables. The shape of the dose-response associations between BMI or waist circumference and hormone concentrations were examined using restricted spline models with knots corresponding to the 10^th^, 50^th^, and 90^th^ percentiles of the sex-specific BMI and waist circumference distributions. Statistical significance for interactions between any two factors was tested using the likelihood ratio test comparing a model with the cross-product term to one without. All statistical tests were two-sided, and a *P*-value of <0.05 was considered statistically significant.

## Results

The mean age of both men and women in the study population was 44 (range: 20–80). Men and women were predominantly non-Hispanic white, never smokers, and light or non-drinkers, with a mean BMI of 27.8 kg/m^2^ (range: 15–50). The distributions of BMI and waist circumference for men and women are shown in **Table S2**. Geometric mean values for TSH, fT_3_ and fT_4_ in the analytic population were 1.6 mIU/L, 3.2 pg/mL, and 0.8 ng/dL, respectively.

Unadjusted for other factors, higher TSH levels were associated with older ages, non-Hispanic white race/ethnicity, non-smoking, moderate drinking, and higher BMI and waist circumference (**Table 1**). Higher fT_3_ levels were observed in men and were associated with younger ages, higher BMI and waist circumference, current smoking, and heavier drinking. There was little variation in fT_4_ across all of these characteristics.

After adjusting for age, race, smoking status, history of diabetes, and alcohol intake, positive associations were observed between BMI and serum TSH levels in men and women with increases in TSH for each quartile increase in BMI of 3.8% (95% CI 0.8%, 6.8%; *P*-trend = 0.02) and 4.0% (95% CI 1.6%, 6.5%; respectively, *P*-trend = 0.003) (**Table 2**). Age and smoking status did not statistically significantly modify the association between BMI and TSH in either men or women (data not shown). The associations between TSH and waist circumference were similar compared to the associations between TSH and BMI. For each quartile increase in waist circumference, TSH increased by 3.1% (95% CI 0.6%, 5.6%; *P*-trend = 0.02) in men and 3.6% (95% CI 1.2%, 6.0%; *P*-trend = 0.01) in women (**Table 2**). Age and smoking status did not significantly modify the waist-TSH association (data not shown). The shape of the dose-response associations are shown graphically in **Figures S1 and S2**.

**Table 2:** Association of BMI and waist circumference with natural logarithm-transformed hormone concentrations, NHANES 2007–2008.

	Men		Women	
	% increase^a^	*P*-trend	% increase^a^	*P*-trend
No. participants	n = 1,623		n = 1,491	
**TSH (mIU/L)**				
BMI (per quartile increase)	3.8 (0.8, 6.8)	0.02	4.0 (1.6, 6.5)	0.003
Waist (per quartile increase)	3.1 (0.6, 5.6)	0.02	3.6 (1.2, 6.0)	0.01
**fT_3_ (pg/mL)**				
BMI (per quartile increase)	1.0 (0.5, 1.5)	0.001	1.3 (0.7, 1.8)	<0.001
Waist (per quartile increase)	1.2 (0.8, 1.7)	<0.001	1.2 (0.6, 1.8)	<0.001
**fT_4_ (ng/dL)**				
BMI (per quartile increase)	−0.5 (−1.2, 0.3)	0.21	−0.7 (−1.6, 0.3)	0.14
Waist (per quartile increase)	−0.4 (−1.5, 0.6)	0.40	−0.6 (−1.5, 0.3)	0.19

a Adjusted for age, race, smoking status, and alcohol intake.

BMI and waist circumference were significantly positively associated with fT_3_ levels in men and women (**Table 2**). These associations were of a weaker magnitude compared to the associations for TSH. Every 1-quartile increase in BMI was associated with a 1.0% increase in fT_3_ in men (*P*-trend = 0.001) and a 1.3% increase in fT_3_ in women (*P*-trend<0.001). Every 1-quartile increase in waist circumference was associated with a 1.2% increase in men (*P*-trend<0.001) and 1.2% increase in women (*P*-trend<0.001). In women, the positive association for BMI was significantly stronger among never smokers compared to ever smokers (*P*-interaction = 0.04). No significant interactions were observed for BMI or waist circumference by age (data not shown). The shape of the dose-response associations are shown graphically in **Figures S3 and S4**.

BMI and waist circumference were non-significantly inversely associated with fT_4_ in men and women (**Table 2**). Age and smoking status did not modify these associations (data not shown).

We observed no significant interactions between BMI and waist circumference on levels of TSH, fT_3_, or fT_4_.

As a sensitivity analysis, we additionally restricted the analysis to men and women within the normal range of thyroid autoantibodies (TPOab<9 IU/mL and TgAb<4 IU/mL: n = 1,502 men and n = 1,287 women) yielded similar results for fT_3_ and fT_4_. The results for TSH were slightly weaker among women (per 1-quartile increase in BMI: 3.2% increase; per 1-quartile increase in waist circumference: 3.2% increase) but remained similar among men.

We also restricted the population to the subset with information on fasting glucose levels (787 men and 722 women). We compared the results before and after adjusting for fasting glucose, a potential mediator of the relationship between body fat and thyroid function. Although we generally observed positive associations between fasting glucose and TSH and fT_3_ levels, associations for BMI and waist circumference with TSH and fT_3_ adjusted for fasting glucose were very similar to results unadjusted for fasting glucose, suggesting that the positive associations observed between BMI and waist circumference and TSH and fT_3_ is not completely mediated by insulin resistance.

## Discussion

In this study of euthyroid men and women taken from a large, nationally-representative sample of the civilian, non-institutionalized U.S. population, we observed statistically significant positive associations of serum TSH and, to a lesser degree, fT_3_ with both BMI and waist circumference. The magnitudes of the associations were generally similar between men and women. We found no association between BMI or waist circumference and fT_4_ levels. In comparing our results to only those other studies that had large sample sizes, adjusted for known confounders (e.g., smoking), and were not restricted to overweight or obese participants or individuals with a medical history of thyroid disease [9]–[11], [16], [18], [19], our finding of a positive association between BMI and TSH is consistent with all but two studies, one of healthy women participating in a primary health screening in Korea [18] and one of adults from rural Western Australia [19].

The basis for the relationship between body fatness and thyroid hormone levels is obscured by the lack of understanding regarding its temporality. In overt hypothyroidism, decreased thyroid hormone actions, particularly those of T_3_, lead to weight gain through reduced basal metabolic rate and decreased physical activity [8]. However, in the euthyroid range of thyroid status, it is unclear whether changes in markers of thyroid function precede changes in weight, or vice versa. Our finding of a positive association of both BMI and waist circumference with TSH and fT_3_ levels is not completely consistent with an influence of thyroid hormones on resting energy expenditure within the euthyroid range. One way in which weight gain could lead to an increase in serum TSH levels and, subsequently, thyroid hormone levels, is through the stimulatory effect of leptin, an adipose tissue-derived hormone, on thyrotropin-releasing hormone (TRH) [25]–[27] or by decreasing thyroid hormone resistance [8]. Other adipokines, such as interleukin-6 and tumor necrosis factor-α, have been suggested to play a role, but their relation to thyroid function remains poorly understood [28]. FT_3_ levels are elevated in obesity to increase resting energy expenditure and prevent additional fat accumulation [8]; thus our finding of a positive association between BMI and waist circumference with fT_3_ may be the result of recent weight gain at the time of blood draw leading to an increase in fT_3_ levels. Interestingly, the same positive association was not observed for fT_4_. One explanation is that the divergent associations for fT_3_ and fT_4_ reflect an increased deiodinase activity in obesity [29]. The positive associations for both TSH and fT_3_ and slightly inverse association for fT_4_, therefore, may reflect mechanisms whereby body fat influences thyroid hormone levels within the euthyroid range.

The major strengths of this study were its large size, which allowed for relatively precise evaluation of the associations of interest, and the survey design, in which participants were sampled to be representative of the entire non-institutionalized U.S. population, possibly yielding more widely representative results than that of many previous studies on this topic. Due to the cross-sectional design of the study, we were limited in our ability to assess the timing of the association between the accumulation of body fat and variation in TSH, fT_3_, or fT_4_ levels. We also lacked data on serum leptin concentrations to directly assess its role in the body fat-TSH association. Waist circumference, which is more strongly predictive of insulin resistance, diabetes, and other cardiometabolic abnormalities compared to BMI [30]–[32], was generally slightly less strongly associated with TSH compared to BMI, suggesting that insulin resistance is unlikely to account for the associations we observed between body fatness and TSH; this was supported by the finding that adjusting for fasting glucose levels in the subset of the population with this information did not attenuate any of the results. However, neither BMI nor waist circumference can distinguish visceral from subcutaneous adipose tissue in the abdomen, which show clear differences with regard to metabolic and lipolytic activity and insulin sensitivity [33]. Thyroid function may need to be evaluated in relation to more accurate measures of visceral and subcutaneous adipose tissue to further understand the biological mechanisms underlying the positive associations between overall and central adiposity and TSH and fT_3_ observed in this and other studies.

In summary, we found that measures of overall and central adiposity were associated with higher circulating levels of TSH and fT_3_ in euthyroid adults. We observed no association with fT_4_ levels. Although weight loss and weight gain are well-known consequences of overt thyroid dysfunction, our results suggest that, within the euthyroid range, excess body weight may induce changes in thyroid hormone levels. Experimental and/or longitudinal studies are needed to assess whether weight loss or maintenance among individuals who are euthyroid may help to prevent the development of subclinical or overt hypothyroidism and associated health risks [34]–[36].

## Supporting Information

Figure S1 **Association between BMI and mean TSH levels in euthyroid men (n = 1,623) and euthyroid women (n = 1,491), NHANES 2007–2008.** Models used restricted quadratic splines and were adjusted for age, smoking status, race/ethnicity, and alcohol intake.
(DOC)

Figure S2 **Association between waist circumference and mean TSH levels in euthyroid men (n = 1,623) and euthyroid women (n = 1,491), NHANES 2007–2008.** Models used restricted quadratic splines and were adjusted for age, smoking status, race/ethnicity, and alcohol intake.
(DOC)

Figure S3 **Association between BMI and mean fT_3_ levels in euthyroid men (n = 1,623) and euthyroid women (n = 1,491), NHANES 2007–2008.** Models used restricted quadratic splines and were adjusted for age, smoking status, race/ethnicity, and alcohol intake.
(DOC)

Figure S4 **Association between waist circumference and mean fT_3_ levels in euthyroid men (n = 1,623) and euthyroid women (n = 1,491), NHANES 2007–2008.** Models used restricted quadratic splines and were adjusted for age, smoking status, race/ethnicity, and alcohol intake.
(DOC)

Table S1 Distribution of thyroid measures in men (n = 2,910) and women (n = 3,025) ages 20+, NHANES 2007–2008.
(DOC)

Table S2 Quartile cutpoints for anthropometric variables in euthyroid men (n = 1,623) and euthyroid women (n = 1,491), NHANES 2007–2008.
(DOC)
