# Supplementary figures and images for: Body Fatness and Markers of Thyroid Function among U.S. Men and Women
Source: PLoS One. 2012 Apr 12;7(4):e34979. doi: 10.1371/journal.pone.0034979 (PMC3325258; doi:10.1371/journal.pone.0034979)

Figure S1.


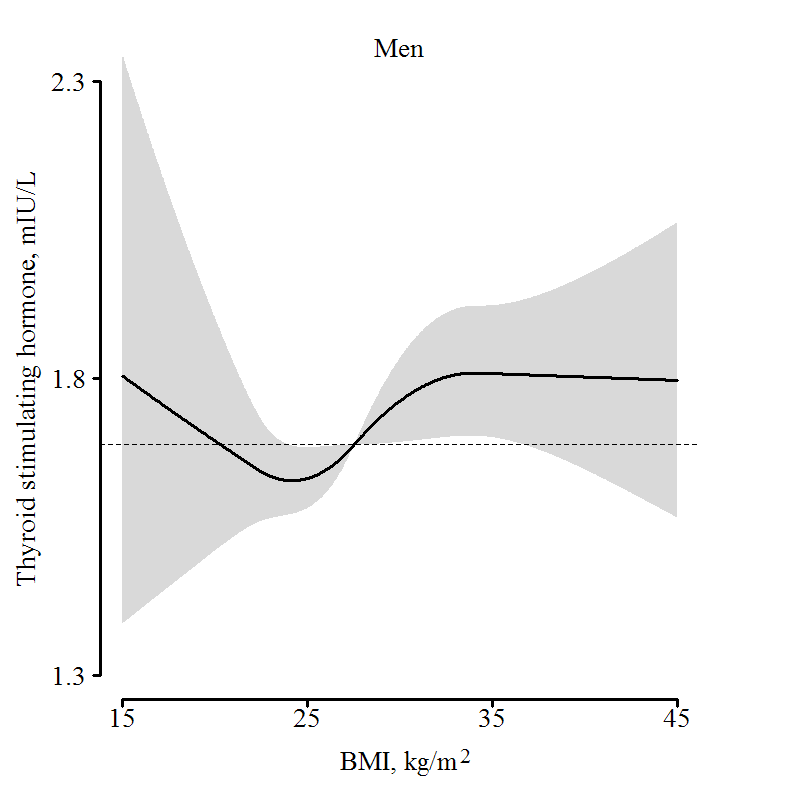

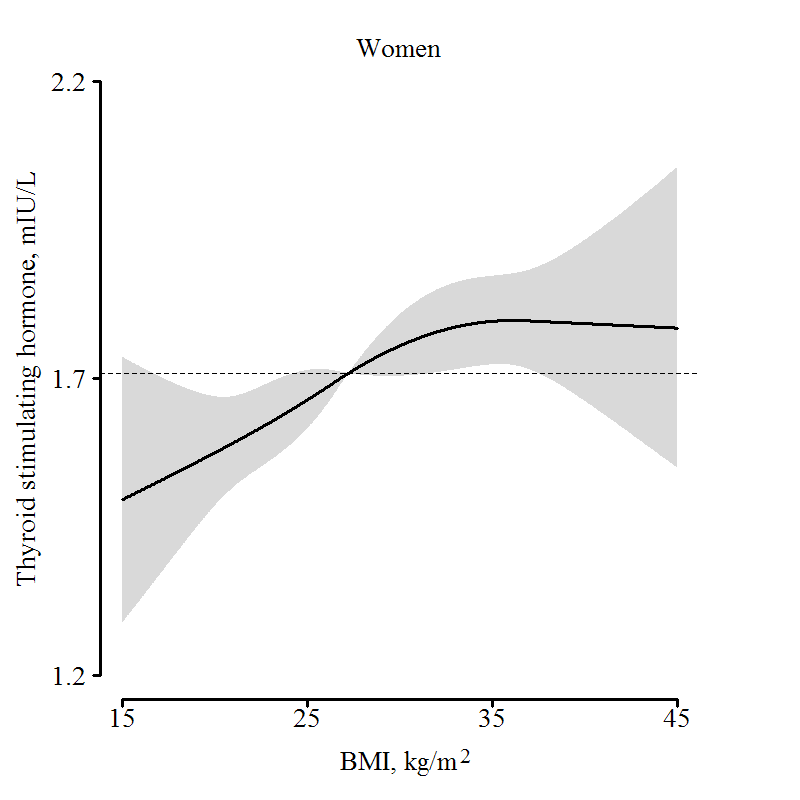

Supplement: Figure S1 — Association between BMI and mean TSH levels in euthyroid men (n = 1,623) and euthyroid women (n = 1,491), NHANES 2007–2008. Models used restricted quadratic splines and were adjusted for age, smoking status, race/ethnicity, and alcohol intake. (DOC) [file pone.0034979.s001.doc]

Figure S2.


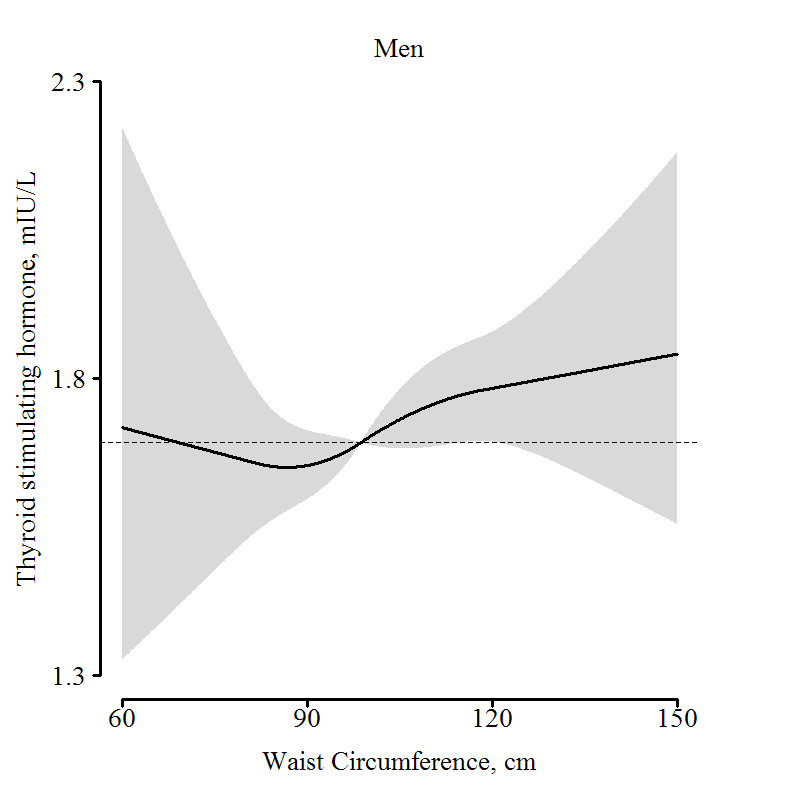

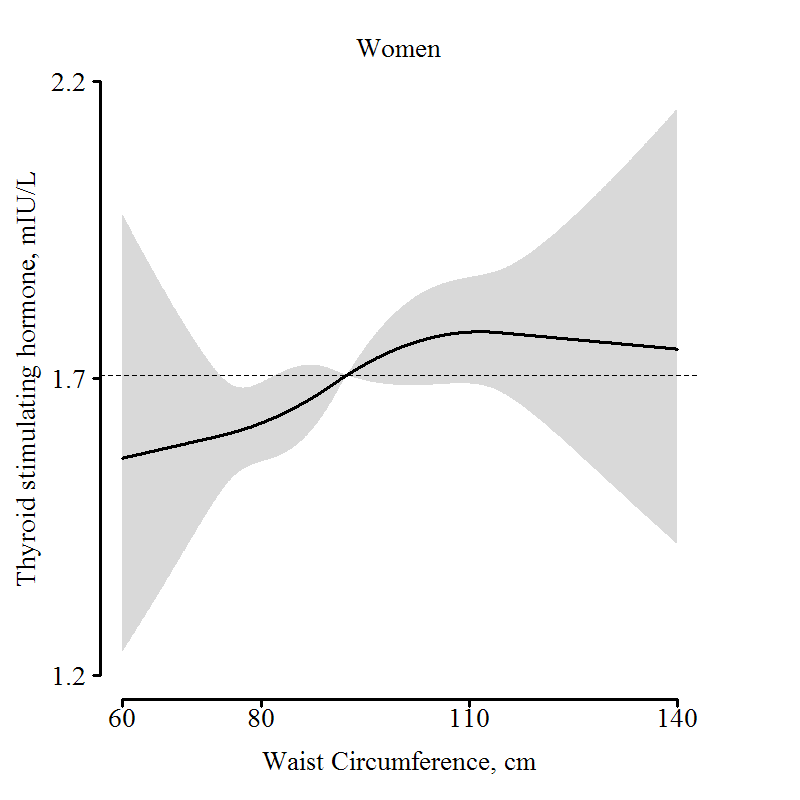

Supplement: Figure S2 — Association between waist circumference and mean TSH levels in euthyroid men (n = 1,623) and euthyroid women (n = 1,491), NHANES 2007–2008. Models used restricted quadratic splines and were adjusted for age, smoking status, race/ethnicity, and alcohol intake. (DOC) [file pone.0034979.s002.doc]

Figure S3.


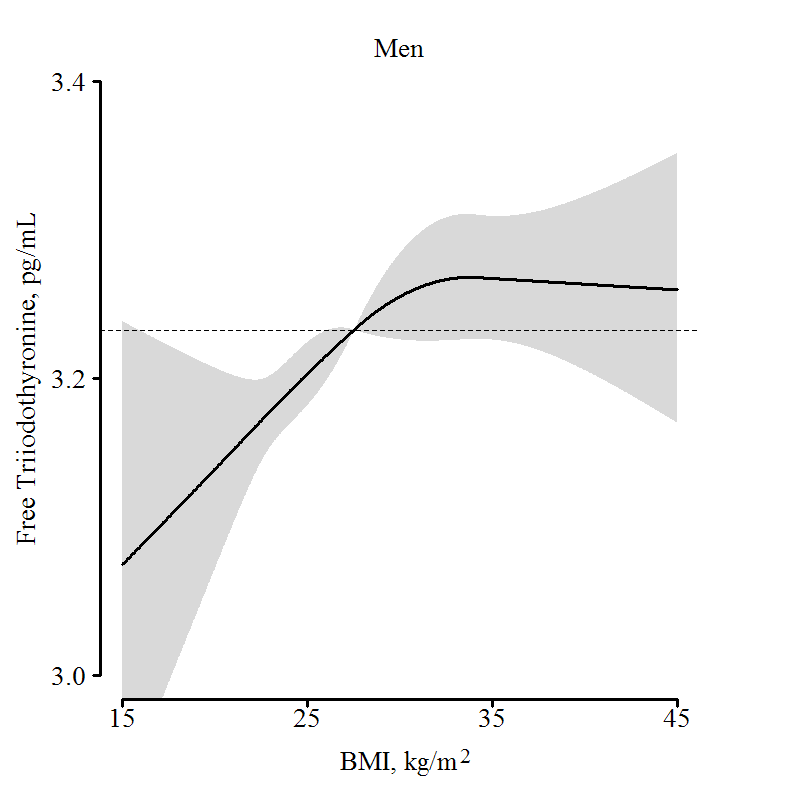

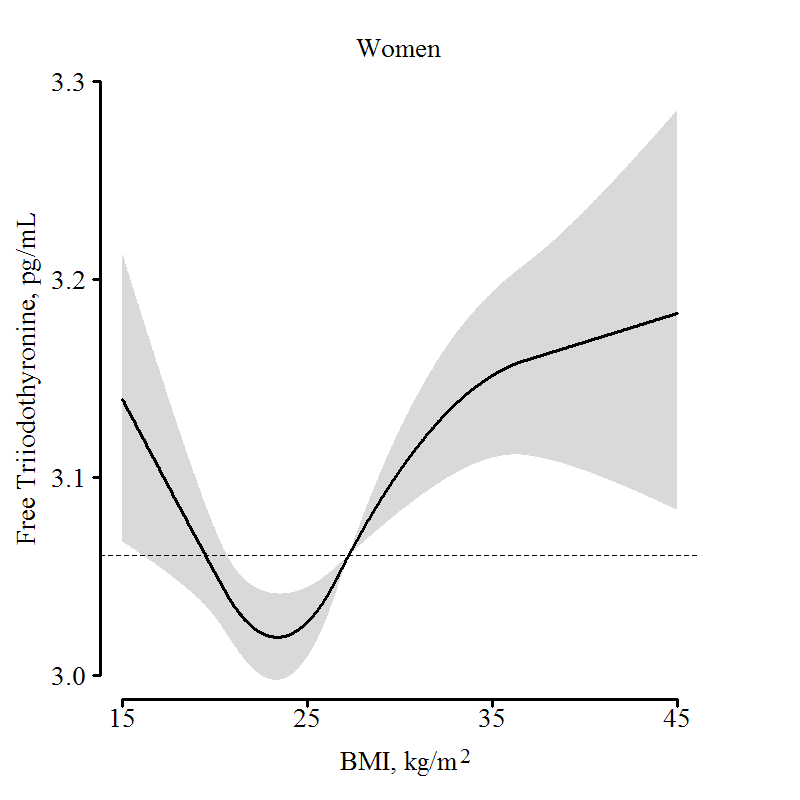

Supplement: Figure S3 — Association between BMI and mean fT3 levels in euthyroid men (n = 1,623) and euthyroid women (n = 1,491), NHANES 2007–2008. Models used restricted quadratic splines and were adjusted for age, smoking status, race/ethnicity, and alcohol intake. (DOC) [file pone.0034979.s003.doc]

Figure S4.


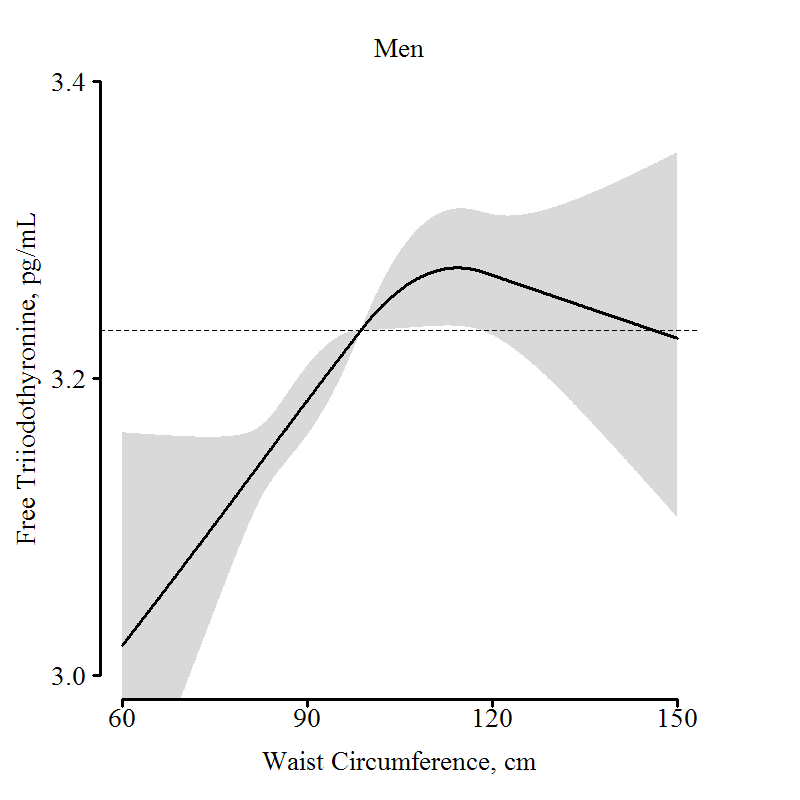

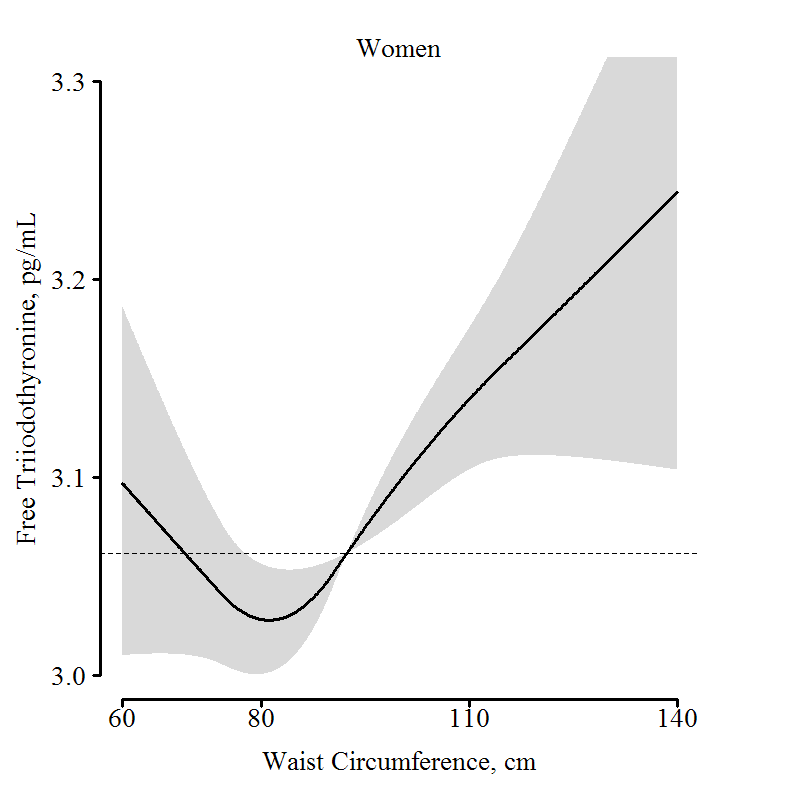

Supplement: Figure S4 — Association between waist circumference and mean fT3 levels in euthyroid men (n = 1,623) and euthyroid women (n = 1,491), NHANES 2007–2008. Models used restricted quadratic splines and were adjusted for age, smoking status, race/ethnicity, and alcohol intake. (DOC) [file pone.0034979.s004.doc]
