# Supplementary material for: Body Fatness and Markers of Thyroid Function among U.S. Men and Women
Source: PLoS One. 2012 Apr 12;7(4):e34979. doi: 10.1371/journal.pone.0034979 (PMC3325258; doi:10.1371/journal.pone.0034979)
Supplement: Table S1 — Distribution of thyroid measures in men (n = 2,910) and women (n = 3,025) ages 20+, NHANES 2007–2008. (DOC) [file pone.0034979.s005.doc]

Table S1.

|  |  | **Men** | | | |  | **Women** | | | |
| --- | --- | --- | --- | --- | --- | --- | --- | --- | --- | --- |
|  | **Units** | **Geometric mean** | **2.5 percentile** | **Median** | **97.5 percentile** |  | **Geometric mean** | **2.5 percentile** | **Median** | **97.5 percentile** |
| **TSH** | mIU/L | 1.62 | 0.42 | 1.61 | 5.81 |  | 1.57 | 0.22 | 1.61 | 6.58 |
| **FT_3_** | pg/mL | 3.26 | 2.5 | 3.2 | 4.2 |  | 3.05 | 2.4 | 3.0 | 4.0 |
| **FT_4_** | ng/dL | 0.77 | 0.6 | 0.8 | 1.2 |  | 0.77 | 0.5 | 0.8 | 1.3 |
| **TPOab** | IU/mL | 0.87 | 0.1 | 0.6 | 117.3 |  | 1.35 | 0.1 | 0.7 | 379.9 |
| **TgAb** | IU/mL | 0.77 | 0.6 | 0.6 | 28.9 |  | 0.99 | 0.6 | 0.6 | 121 |
