# Supplementary material for: Body Fatness and Markers of Thyroid Function among U.S. Men and Women
Source: PLoS One. 2012 Apr 12;7(4):e34979. doi: 10.1371/journal.pone.0034979 (PMC3325258; doi:10.1371/journal.pone.0034979)
Supplement: Table S2 — Quartile cutpoints for anthropometric variables in euthyroid men (n = 1,623) and euthyroid women (n = 1,491), NHANES 2007–2008. (DOC) [file pone.0034979.s006.doc]

Table S2.

|  | **Men** | | | |  | **Women** | | | |
| --- | --- | --- | --- | --- | --- | --- | --- | --- | --- |
|  | **Quartile 1** | **Quartile 2** | **Quartile 3** | **Quartile 4** |  | **Quartile 1** | **Quartile 2** | **Quartile 3** | **Quartile 4** |
| **Body mass index (kg/m^2^)** |  |  |  |  |  |  |  |  |  |
| Range | 16.9-24.5 | 24.5-27.5 | 27.6-30.9 | 30.9-48.9 |  | 15.8-23.5 | 23.5-27.3 | 27.3-31.8 | 31.9-49.8 |
| Median | 22.6 | 26.2 | 29.0 | 33.7 |  | 21.4 | 25.4 | 29.3 | 36.3 |
| **Waist circumference (cm)** |  |  |  |  |  |  |  |  |  |
| Range | 67.8-90.2 | 90.3-98.7 | 98.8-107.8 | 107.9-150.0 |  | 61.6-83.4 | 83.5-92.4 | 92.5-103.2 | 103.5-142.2 |
| Median | 83.8 | 94.8 | 103.4 | 116.1 |  | 77.5 | 87.8 | 97.6 | 112.4 |
